# Correction: Characterization of Carriage Isolates of *Neisseria meningitidis* in the Adolescents and Young Adults Population of Bogota (Colombia)

**DOI:** 10.1371/journal.pone.0139983

**Published:** 2015-10-05

**Authors:** The PLOS ONE Staff

## Notice of Republication

This article was republished on September 21, 2015, to correct an error in the title that was introduced during production of the article: *meningitides* incorrectly read *meningitides*. The publisher apologizes for the error. Please view the originally published, uncorrected article and the republished, corrected article here.

## Supporting Information

S1 File Originally published, uncorrected article. (PDF)

S2 File Republished, corrected article. (PDF)
