# Supplementary material for: Characterization of Carriage Isolates of Neisseria meningitidis in the Adolescents and Young Adults Population of Bogota (Colombia)
Source: PLoS One. 2015 Aug 31;10(8):e0135497. doi: 10.1371/journal.pone.0135497 (PMC4556189; doi:10.1371/journal.pone.0135497)
Supplement: S1 Table — (DOC) [file pone.0135497.s001.doc]

# S1Table. Factors associated with the carriage of *N. meningitides*

|  | **Non carrier** | | **Carrier** | | ***x*^2^ Test** |
| --- | --- | --- | --- | --- | --- |
|  | **n** | **%** | **n** | **%** |  |
| **Gender** | | | | | |
| Female | 787 | 93.58 | 54 | 6.42 | 0.445 |
| Male | 572 | 92.56 | 46 | 7.44 | - |
| **Intimate kissing** | | | | | |
| Yes | 902 | 92.51 | 73 | 7.49 | 0.174 |
| No | 457 | 94.42 | 27 | 5.58 | - |
| **Attendance to social venues** | | | | | |
| Yes | 1074 | 92.67 | 85 | 7.33 | 0.154 |
| No | 285 | 95.00 | 15 | 5.00 | - |
| **Exposure to cigarette smoke at home** | | | | | |
| Yes | 493 | 91.81 | 44 | 8.19 | 0.122 |
| No | 866 | 93.93 | 56 | 6.07 | - |
| **Oral sex** | | | | | |
| Yes | 233 | 89.96 | 26 | 10.04 | **0.025** |
| No | 1126 | 93.83 | 74 | 6.17 | - |
| **Smoking** | | | | | |
| Yes | 189 | 91.30 | 18 | 8.70 | 0.258 |
| No | 1170 | 93.45 | 82 | 6.55 | - |
| **Antibiotic consumption (previous month)** | | | | | |
| Yes | 189 | 91.5 | 17 | 8.25 | 0.391 |
| No | 170 | 93.38 | 83 | 6.62 | - |
| **Biomedical studies** | | | | | |
| Yes | 141 | 92.16 | 12 | 7.84 | 0.546 |
| No | 747 | 93.49 | 52 | 6.51 | - |
| **Share bedroom** | | | | | |
| Yes | 449 | 92.39 | 37 | 7.61 | 0.417 |
| No | 910 | 93.53 | 63 | 6.47 | - |
| **Respiratory infection (previous month)** | | | | | |
| Yes | 445 | 93.10 | 33 | 6.90 | 0.958 |
| No | 914 | 93.17 | 67 | 6.83 | - |
| **Previous meningococcal vaccine** | | | | | |
| Yes | 8 | 100 | 0 | 0 |  |
| No | 1351 | 93.10 | 100 | 6.09 |  |
