# Supplementary material for: Correction: Characterization of Carriage Isolates of Neisseria meningitidis in the Adolescents and Young Adults Population of Bogota (Colombia)
Source: PLoS One. 2015 Oct 5;10(10):e0139983. doi: 10.1371/journal.pone.0139983 (PMC4593576; doi:10.1371/journal.pone.0139983)
Supplement: S2 File — (PDF) [file pone.0139983.s002.pdf]

PLOS ONE

RESEARCH ARTICLE

Characterization of Carriage Isolates of *Neisseria meningitidis* in the Adolescents and Young Adults Population of Bogota (Colombia)

Jaime Moreno[1]*, Melissa Hidalgo[1], Carolina Duarte[1], Olga Sanabria[1], Jean Marc Gabastou[2], Ana Belén Ibarz-Pavon[2]

1 Grupo de Microbiología, Instituto Nacional de Salud, Bogotá, Colombia, **2** Pan-American Health Organization, Washington DC, United States of America

* jmoreno@ins.gov.co

OPEN ACCESS

Citation: Moreno J, Hidalgo M, Duarte C, Sanabria O, Gabastou JM, Ibarz-Pavon AB (2015) Characterization of Carriage Isolates of *Neisseria meningitidis* in the Adolescents and Young Adults Population of Bogota (Colombia). PLoS ONE 10(8): e0135497. doi:10.1371/journal.pone.0135497

Editor: Caroline L Trotter, University of Cambridge, UNITED KINGDOM

Received: November 12, 2014

Accepted: July 22, 2015

Published: August 31, 2015

Copyright: © 2015 Moreno et al. This is an open access article distributed under the terms of the Creative Commons Attribution License, which permits unrestricted use, distribution, and reproduction in any medium, provided the original author and source are credited.

Data Availability Statement: All relevant data are within the paper and its Supporting Information file.

Funding: This study was supported by Sanofi-Pasteur, through the Pan-American Health and Education Foundation (PAHEF). The funders had no role in study design, data collection and analysis, decision to publish, or preparation of the manuscript.

Competing Interests: Funding to the Pan-American Health Organization was provided by Sanofi-Pasteur through the Pan-American Health Organization Foundation. There are no patents, products in

Abstract

Background

Meningococcal carriage studies are important to improve our understanding of the epidemiology of meningococcal disease. The aim of this study was to determine the prevalence of meningococcal carriage and the phenotypic and genotypic characteristics of isolates collected from a sample of students in the city of Bogotá, Colombia.

Materials and Methods

A total of 1459 oropharyngeal samples were collected from students aged 15–21 years attending secondary schools and universities. Swabs were plated on a Thayer Martin agar and *N. meningitidis* was identified by standard microbiology methods and PCR.

Results

The overall carriage prevalence was 6.85%. Carriage was associated with cohabitation with smokers, and oral sex practices. Non-groupable and serogroup Y isolates were the most common capsule types found. Isolates presented a high genetic diversity, and circulation of the hypervirulent clonal complexes ST-23, ST-32 and ST-41/44 were detected.

Conclusion

The meningococcal carriage rate was lower than those reported in Europe and Africa, but higher than in other Latin American countries. Our data also revealed antigenic and genetic diversity of the isolates and the circulation of strains belonging to clonal complexes commonly associated with meningococcal disease.

development or marketed products to declare. This does not alter the authors' adherence to all the PLOS ONE policies on sharing data and materials. Additionally, the authors would like to point out that there was no direct contact between the funders and the authors.

Introduction

Neisseria meningitidis, the meningococcus, is a harmless commensal of the human nasopharynx. The carrier state is usually asymptomatic and essential for transmission. Invasion of the pharyngeal tissues and subsequent proliferation in the blood and cerebrospinal fluid are infrequent, but lead to life-threatening diseases such as septicemia and meningitis, which are associated with significant levels of morbidity and mortality [1].

Asymptomatic carriage is an age-dependent phenomenon, with point prevalence carriage rates usually ranging from 10% to 35% in young adults. It is likely that, at one time or another during life, most individuals are colonized with meningococci [2]. Despite high rates of meningococcal carriage, the disease is rare, with annual incidence varying from 1 to 1000 cases per 100,000 individuals in different parts of the world [3]. In Colombia, the annual incidence rate of meningococcal disease varies from 0.55 in 1994 to 1.02 in 2011 cases per 100,000 inhabitants [4], and *N. meningitides* serogroup B isolates were the most frequently received by the National Reference Laboratory [5].

Horizontal genetic exchange and recombination events in the genome of *N. meningitidis* during residence in the human nasopharynx result in antigenic diversity, even within closely-related strains that belong to the same clonal complexes. As a result, individual clones may express themselves in more than one serogroup. Isolates of *N. meningitidis* obtained from disease cases tend to be associated with a limited range of clonal complexes that are referred as hypervirulent lineages. In contrast, isolates from carriers are genetically more heterogenous and relatively few belong to hyperinvasive clones [6].

Studies of *N. meningitidis* isolated from the nasopharynx are important to improve our understanding of the bacterium's population structure and dynamics, and contribute to the detection of hyper-invasive strains which may vary in time and among geographic regions. These studies are also important to establish carriage rates among different populations, allowing the identification of risk groups that could eventually be targeted in a vaccination campaign. Additionally, carriage studies that include molecular characterization should be performed to identify the emergence of hypervirulent strains [2–3,6,7]. The aim of this study was to determine the prevalence of meningococcal carriage and the phenotype and genotype characteristics of isolates collected from a sample of students in the city of Bogotá (Colombia). The study was performed within the frame of the research program for Improving Surveillance and Characterization of Meningococcal Disease in Latin America and The Caribbean Region, which was supported by Surveillance System for the Bacterial Agents Responsible for Pneumonia and Meningitis (SIREVA II) program of The Pan American Health Organization (PAHO).

Materials and Methods

Study population

The study was carried out between August and October 2012. Students aged 15–21 years attending secondary schools (n = 12) and universities (n = 7) in Bogotá (Colombia) were enrolled in the study. Sample size was calculated on the assumption of a total population of 600,000 students aged between 15 and 21 years. Based on published data, carriage prevalence was estimated at 18%, and we allowed for a 15% non-participation. Calculations were performed for a 95% confidence level and desired precision of 2%. The calculated study population size was 1377. In order to maximize participation, all of the children of 9th to 11th scholar grade were invited by giving detailed explanations concerning *N. meningitidis* carriage and its related diseases. Parents of the school children were informed in writing about the purpose and procedure of the study, and were asked to give informed consent for their child's participation

in this research. All the children whose parents permitted the students to take part in the study were included. University students healthy were enrolled based on their consent to participate in the study. A written informed consent form was signed by each participant, and consent was also obtained from parents/guardians of subject aged less than 18 years. Before the sample collection, the students answered a structured questionnaire that included information on demographic data and known risk factors for *N. meningitidis* carriage. Oro-pharyngeal swab samples through the mouth were taken by trained staff from the posterior wall of the oropharynx and were immediately placed in Culture Swab Plus—AMIES coal, and transported within 4 hours to the Microbiology Group at the Instituto Nacional de Salud (INS) for identification, and phenotypic and genotypic characterization.

Ethical approval

The study was approved by the Comité de Etica en Investigación (CEIN) of the Instituto Nacional de Salud and by Pan American Health Organization's Ethics Review Committee's (PAHOERC)

Isolation of *N. meningitidis*

Swabs were immediately plated on a Thayer Martin agar and incubated at 37°C with 5% CO_2 atmosphere for 72 hours. Morphological evaluation of bacterial colonies was performed and one colony sub-cultured and identified by standard methods of colony morphology, Gram stain, oxidase, biochemical profile using VITEK automated identification, and by PCR targeting *crg*A gene [8].

Characterization of meningococcal isolates

Serogroup was determined by slide agglutination with commercial antiserum against meningococcal capsular polysaccharides (DIFCO, Beckton Dickinson), and confirmed by PCR using specific amplification of the different genes corresponding to serogroups A, B, C, Y, and W [8,9] Those isolates for which the PCR rendered a negative result were tested by PCR for the presence of the capsule null (*cnl*) region [10]. Serotype and serosubtype were determined by dot blot with monoclonal antibodies (RIVM, Bilthoven, the Netherlands, and Institute Adolfo Lutz, São Paulo, Brazil) [11]. MLST was performed at the INEI-ANLIS "Dr. Carlos G. Malbrán" Laboratory in Buenos Aires (Argentina), according to the method described by Maiden et al. [12], and sequence types (STs) were assigned according to information available at the Neisseria MLST website (http://pubmlst.org/neisseria).

Statistical analysis

Initial descriptive data analyses were performed in Microsoft Excel. Risk factors of carriers and non-carriers were initially assessed by single-variable logistic regression, and by a multilevel variable analysis stratified by oral sex practice, since it was the only risk factor that rendered a significant p-value in the univariable test. All logistic regression tests were performed using STATA/SE 12 for MacOS.

Results

A total of 1459 oropharyngeal samples were collected, and 100 *N. meningitidis* were isolated, rendering an overall carriage rate of 6.85%. Additionally, other *Neisseria* species were identified: *N. lactamica* (n = 22; 1.5%), *N. elongata* (n = 7; 0.5%) and *N. cinerea* (n = 1; 0.06%). *N.*

Table 1. Distribution of the percentage of *N. meningitidis* carriers by age.

Students		*N. meningitidis*		Other *Neisserias**	
Age (Years)	n	n	%	n	%
15	186	8	4.3	6	3.2
16	192	16	4.2	7	3.6
17	221	11	3.6	7	3.2
18	322	17	2.5	2	0.6
19	250	26	3.2	6	2.4
20	174	13	4.6	0	0.0
21	114	9	7.0	2	1.8
**Total**	1459	100	6.85	30	2.05

**N. lactamica* (n = 22), *N. elongata* (n = 7) and *N. cinerea* (n = 1).

doi:10.1371/journal.pone.0135497.t001

meningitidis carriage rate peaked among those aged 20 (4.6%) and 21 (7.0%) years of age (Table 1).

The distribution of demographic data and factors associated with *N. meningitidis* carriage of participants are shown in S1 Table. An initial Chi-square analysis showed no differences in carriage for gender, intimate kissing, attendance to social venues, direct or indirect exposure to cigarette smoke, sharing the bedroom, biomedical science students, respiratory infection and antibiotic consumption on the previous month (S1 Table). However, a statistically significant difference was detected among those who engaged on oral sex practices (p = 0.025). In a univariate logistic regression analysis, male gender, being aged over 17 years, intimate kissing, attendance to social venues, and exposure to cigarette smoke (both direct and indirect) were all positively associated with meningococcal carriage, although these associations were not statistically significant. Practicing oral sex, however, presented the strongest association and was significant, although this significance was not sustained in the multivariable analysis adjusting for gender, intimate kissing, attendance to social venues, sharing the bedroom or smoking (data not shown). Stratified analysis adjusting for oral sex practices showed the results among those who practice oral sex were not significantly different from those obtained among those who did not, even though males were more likely to engage this practice than females (Table 2).

Using the PCR methodology, 32 isolates presented the *cnl* locus, and 25 were non-groupable. Group Y was identified in 22 isolates, of which 10 (45.5%) were expressing the capsule. A total of 15 isolates were group B, of which 7 (46.6%) were capsulated. All 3 serogroup C isolates were capsulated, and one of three W also expressed the capsule (Fig 1). A total of 31 different serotype:serosubtype combinations were found. The combination NT:NST was common among NG and groups C and W isolates. The combination 14:NST was mainly associated to group Y isolates. NG isolates were predominant among the combinations 15:P1.16 (7.0%) and NT:P1 (Table 3).

Sequence type was determined for 74 of the 100 meningococcal isolates. Overall, 72 isolates were grouped into one of 14 clonal complexes with different serotype:serosubtype combinations (Table 3). A total of 16 isolates (21.6%) belonged to the ST-23 clonal complex, of which 14 were group Y, and 12 presented the phenotype Y:14:NST. Most of the NG isolates were grouped into clonal complexes ST-53 (n = 13), ST-198 (n = 10), ST-1136 (n = 6) and ST-178 (n = 4). Group B isolates were distributed into the ST41/44, ST-32, ST-162 and ST-231 clonal complexes. Group W isolates belonged to the ST-22 complex, and two group C isolates, together with three NG were grouped into the ST-35 clonal complex (Fig 2).

Table 2. Association between risk factors and meningococcal carriage.

Risk factor	Total included	Carriers			Non carriers			OR	[CI]	p value	Adjusted OR by oral sex practice	[CI]	p value
		Total	Exposed	%	Total	Exposed	%						
Gender*	1459	100	46	46.00	1359	572	42.09						
Age group**	1459	100	65	65.00	1359	795	58,5	1.32	[0.85–2.08]	0.198	1.20	[0.78–1.85]	0.132
Intimate kissing	1459	100	73	73.00	1359	902	66.37	1.37	[0.86–2.25]	0.174	1.22	[0.76–1.96]	0.541
Attendance to social venues	1459	100	85	85.00	1359	1074	79.03	1.50	[0.85–2.85]	0.154			
Exposure to cigarette smoke at home	1459	100	44	44.00	1359	493	36.28	1.38	[0.89–2.12]	0.122	1.34	[0.89–2.02]	0.372
Smoking	1459	100	18	18.00	1359	189	13.91	1.36	[0.75–2.35]	0.258	1.23	[0.71–2.13]	0.571
Oral sex	1459	100	26	26.00	1359	233	17.14	1.70	[1.02–2.75]	0.030			
Biomedical studies***	952	64	12	18.75	888	141	15.88	1.22	[0.58–2.39]	0.546	1.22	[0.64–2.35]	0.203
Sharing the bedroom	1459	100	37	37.00	1359	449	33.04	1.19	[0.76–1.85]	0.417	1.21	[0.79–1.85]	0.177
Respiratory infection (previous months)	1459	100	33	33.00	1359	445	32.74	1.01	[0.64–1.58]	0.958	0.99	[0.64–1.53]	0.871

* Exposed = male.

** Groups defined 18-21years.

***Only those attending university were included for this test.

doi:10.1371/journal.pone.0135497.t002

Discussion

Meningococcal carriage prevalence has been found to be variable within and between countries, and across age groups. Likewise, serogroup distribution is also unique to geographic

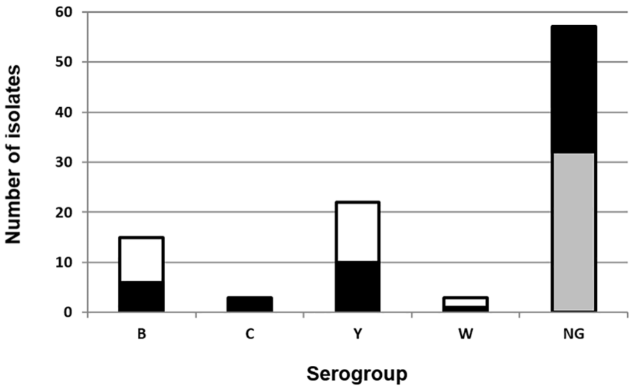

Fig 1. Identification of serogroups of N. meningitidis with conventional slide agglutination and detection of capsule null (cnl) region. The figure represents the number of isolates of *N. meningitidis* by serogroup. Capsulated isolates for each serogroup are represed in black, whereas non-capsulated appear in white. For the NG, those presenting the *cnl* insertion appear in grey, and those that could not be serogrouped by either PCR or agglutination are represented in black.

doi:10.1371/journal.pone.0135497.g001

Table 3. Association of serogroup, serotype and subtypes with MLST clonal complexes identified in this study.

Clonal complex	n	%	Serogroup:Serotype:Subtypes
ST-23 complex	16	21.6	Y:14:NST (12), Y:14:P1.5 (1), Y:NT:P1.5,2 (1), NG:NT:NST (2)
ST-53 complex	13	16.6	NG:NT:NST (7), NG:NT:P1.7 (5), NG:NT:P1.9 (1)
ST-198 complex	10	13.5	NG:15:NST (3), NG:15:P1.16 (3), NG:15:P1.7 (3), NG:14:P1.16 (1)
ST-1136 complex	6	8.1	NG:15: P1.1,16 (2), NG:15:P1.7 (2), NG:15:NST (1), NG:15:P1.7,16 (1)
ST-35 complex	5	6.8	C:NT:NST (2), NG:4,7:NST (1), NG:4,7:P1.13 (1), NG:4:P1.16 (1),
ST-178 complex	4	5.4	NG:NT:NST (4)
ST-167 complex	3	4.1	Y:10,14,17:P1.5 (1), Y:14:P1.5(1), Y:4,7:P1.5 (1)
ST-22 complex	3	4.1	W:NT:NST(3)
ST-41/44 complex	3	4.1	B:1:P1.22.1(1), B:10:NST(1), B:4:P1.16 (1)
ST-1157 complex	2	2.7	NG:4:P1.22.1,9 (1), NG:NT:P1.14 (1)
ST-32 complex	2	2.7	B:4,7:NST (1), B:7,9:NST (1)
ST-60 complex	2	2.7	NG:NT:NST (2)
New ST	2	2.7	Y:14:P1.4 (1), NG:7,17:P1.22.1 (1)
ST-162 complex	1	1.4	B:NT:P1.12 (1)
ST-213 complex	1	1.4	B:10:P1.7 (1)
ST-750 complex	1	1.4	B:NT:P1.22.1,1 (1)

doi:10.1371/journal.pone.0135497.t003

regions, and can change over time [13]. In this study, we present the characteristics of meningococcal carriage isolates collected in students aged 15–21 years in Bogotá, Colombia. The results show that the overall carriage prevalence was 6.85%, which is lower than the average 10–35% reported in carriage studies conducted in Europe and Africa [13,14], but yet higher than that reported recently from Santiago, (4.0%) Chile [15] and Mexico (1.6%) [16]. These lower carriage rates found in our study and those conducted in Mexico and, more recently, Chile, could be one of the reasons why disease incidence is lower in these countries than those usually reported among high and middle-income countries. It is worth noticing that Colombia and Chile have well-established national epidemiological and reporting surveillance systems and therefore, their reported disease incidence rates can be considered accurate.

Asymptomatic pharyngeal colonization of *N. meningitidis* is known to be an age-dependent phenomenon. A systematic review and meta-analysis of meningococcal carriage studies found that, the carriage increasing from 4.5% in infants to 23.7% in 19-year olds and decreasing to 7.8% in adults over 50 years of age [17]. A study conducted among students from 15 to 19

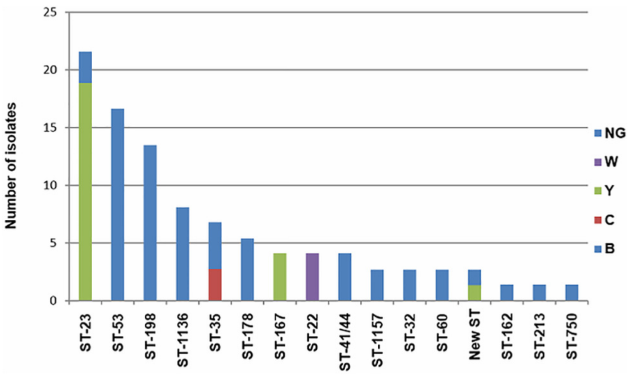

Fig 2. Distribution of serogroups according to *N. meningitidis* clonal complex.

doi:10.1371/journal.pone.0135497.g002

years of age recruited from 74 schools or colleges in United Kingdom, the overall frequency of carriage was 16.7% [18], and a cross sectional study analyzing pharyngeal swabs from among students at Nottingham University, the carriage rates increased from 23.2% in first-year students to 34.2% among second-year students [19]. However, a study conducted among adolescent aged between 15 and 19 years in Mexico City, *N. meningitidis* was isolated from nasopharyngeal exudates in 1.6% of participants [16], indicating that there may be geographic variability of carriage rates in college students. It is worth noticing that whereas in the UK it is common for students to move out of the parental home and into student halls to attend university, this is not an extended practice in Colombia, where university students usually remain at home for the duration of their degree. Therefore, they are not exposed to the closed-quarters and over-crowded conditions that are encountered in student residences. This might also prevent them from being overly exposed to some of the well-known behavior risk factors associated to these circumstances and to meningococcal carriage, such as very frequent attendance to social venues. Nonetheless, the adolescent and young adult population remains crucial on the dissemination and transmission of this pathogen. For this reason, the Advisory Committee on Immunization Practices (ACIP-USA) recommend including meningococcal immunization to all persons aged 11–18 years as an effective strategy to reduce meningococcal disease incidence among adolescents and young adults [20,21].

Harrison, *et al*., indicated that the incidence of meningococcal infection in college students is similar to the incidence in the general population of the same age [22]. Therefore, the lower carriage rate found in our study in Bogotá could explain the lower incidence of meningococcal disease in Colombia. The capital city, with 7.8 million inhabitants, is the most populated city in the country, and according to data from SIREVA II, only six isolates were recovered from patients with invasive meningococcal disease in 2012 [5].

The swabbing technique and culture conditions are factors that influence the detection of meningococcal carriage in individuals. In this study, samples were taken from the posterior wall of the oropharynx and were placed in an appropriate transport medium and transported within 4 hours to the laboratory and processed immediately upon arrival. The posterior pharyngeal swabs are significantly better than nasopharyngeal swabs for evaluating *N. meningitidis* carrier status [23]. Caugant DA, *et al*., showed that direct plating was not significantly better than the use of Stuart's transport media, at least when the plating in the laboratory was performed within 4 h of taking the samples [2].

A number of risk factors have been shown to be associated with meningococcal carriage including: male sex, active and passive cigarette smoking, attending pubs and clubs, discotheque visits, antimicrobial drug use, intimate kissing and overcrowding [18,24]. However, achieving statistical significance in such studies is often difficult since meningococcal carriage is low and the number of sampled individuals is often limited due to logistical reasons. This study found a positive association between carriage and the already described risk factors such as age, intimate kissing, attendance to social venues, and direct or indirect exposure to cigarette smoke, although these were not statistically significant. To the authors' knowledge, this is the first study to investigate the association of meningococcal carriage with the practice of oral sex. Despite the limited size of the study population, this association showed statistical significance and demonstrates that oral sex enhances meningococcal transmissibility, which would also maximize the chances of *N. meningitidis* and *N. gonorrhoeae* coming together despite their different habitats. A previous study conducted by Tayal SC, et al., found no relationship of meningococcal carriage with sexual orientation and orogenitaI sex [25]. However, cases of genital tract infections or colonization due to *N. meningitidis* have been reported [26,27], and genetic exchange between these two species is a well-documented phenomenon.

Most *N. meningitidis* isolates obtained in this study were NG. Approximately 40% of strains from carriage studies in Europe are NG [13], while in the meningitis belt lower proportions of NG strains have been reported [28,29]. The loss of expression of capsular polysaccharide enhances the capability of meningococci to colonize the human nasopharynx. Moreover, the colonization with NG isolates may be beneficial to the host by eliciting cross-reactive immune responses to non-capsular meningococcal surface antigens [30]. There are two reasons for the lack of capsule: the deletion of the capsule locus (capsule null) or down-regulation of the expression of the capsule either temporarily or permanently by different genetic mechanisms [2]. The phenotypes and clonal composition of meningococci NG were highly diverse, represented mainly by ST-53, ST-198, ST-1136, ST-178 and ST-35 clonal complexes. Claus H, *et al*., demonstrated that *cnl* sequences can be a stable feature of some meningococcal genotypes, represented by the ST-53, ST-198, ST-1136 and ST-1117 clonal complexes [10].

Group Y isolates were associated with phenotype14:NST and ST-23. Serogroup Y is the third most frequent cause of meningococcal disease in Colombia [5], and an increase in the frequency of this serogroup with serotype 14 was observed between 2003 (20%) to 2006 (50%) [31] and declined to 15.1% in 2012 [5]. Additionally, MLST characterization of isolates obtained during 2000–2006 showed that these belonged to the ST-23 clonal complex [32]. These results demonstrate the circulation of pathogenic serogroup Y in asymptomatic people. Isolates of the ST-23 complex were more likely associated with asymptomatic carriers than as a disease cause [33]. However, serogroup Y disease has been reported in the United States, South America, South Africa and Europe countries associated with ST-23 and ST-167 clonal complexes [34].

Phenotypic expression and clonal association in group B isolates were highly diverse. The genetic and antigenic diversity in serogroup B strains that cause sporadic and outbreaks of disease represents a challenge to control through vaccination [34]. This serogroup has been responsible for most meningococcal disease cases in Colombia. ST-41/44, the most common complex in the Neisseria MLST database (http://pubmlst.org/neisseria/), has been associated with the serogroup B and found in both carrier and invasive isolates [2,13,34]. Strains from the hyperinvasive lineage ST-32 clonal complex are isolated predominantly from disease cases [33,35]. ST-32 also been identified among the meningococci from carriers from in Latin American countries [36,37].

Group W isolates were phenotype NT: NST and ST-22 and in serogroup C isolates ST-35 were identified. Isolates W from ST-22 complex have been associated with carriage while strains from the ST-11 complex were a cause of endemic meningococcal disease [38] and associated with a high mortality rate in South Africa [39], has been noticed in Brazil [40] and was first cause of disease in Argentina in 2010 [41] and Chile during 2012 [42]. There is a strong association between ST-11 complex and the expression of a serogroup C capsule and its pathogenic behavior, in counterbalance of their ability to be carrier for a long time [2].

In conclusion, our data show the circulation of meningococcal isolates belonging to hypervirulent clonal complexes commonly associated with meningococcal disease. Carriage studies remain crucial to improve our understanding of the population dynamics of *N. meningitidis* and the epidemiology of meningococcal disease. Information regarding the molecular epidemiology and evolution of disease, and how that relates to carried meningococci within a given population are central to the design implementation and assessment of menigococcal vaccines [3].

Supporting Information

S1 Table. Factors associated with the carriage of *N. meningitides*.
(DOC)

Acknowledgments

We acknowledge Elizabeth Castañeda (PhD and Emeritus Researcher, Instituto Nacional de Salud, Bogotá, Colombia) and Clara Ínes Agudelo for her critical review to the manuscript. We also thank Lic. Cecilia Sorhouet and Dra Lorena Gaita at the INEI-ANLIS "Dr. Carlos G. Malbrán of Argentina for her assistance with Molecular typing by MLST.

Author Contributions

Conceived and designed the experiments: JMG ABI JM. Performed the experiments: MH OS CD JM. Analyzed the data: JM CD MH ABI. Contributed reagents/materials/analysis tools: JMG ABI CD. Wrote the paper: JM ABI.

References

1. Caugant DA. (2008) Genetics and evolution of *Neisseria meningitidis*: importance for the epidemiology of meningococcal disease. Infect Genet Evol 8:558–65. doi: 10.1016/j.meegid.2008.04.002 PMID: 18479979
2. Caugant DA, Tzanakaki G, Kriz P. (2007) Lessons from meningococcal carriage studies. FEMS Microbiol Rev 31:52–63. PMID: 17233635
3. Caugant DA, Maiden MC. (2009) Meningococcal carriage and disease-population biology and evolution. Vaccine 27 Suppl 2:B64–70. doi: 10.1016/j.vaccine.2009.04.061 PMID: 19464092
4. Pinzón-Redondo H, Coronell-Rodriguez W, Díaz-Martinez I, Guzmán-Corena A, Constenla D, et al. (2014) Estimating costs associated with a community outbreak of meningococcal disease in a Colombian Caribbean city. J Health Popul Nutr 32:539–48. PMID: 25395916
5. Instituto Nacional de Salud. Aislamientos invasores de *Streptococcus pneumoniae Haemophilus influenzae Neisseria meningitidis*. Colombia Resultados de la vigilancia por departamentos SIREVA II 2003–2012. Available: http://www.ins.gov.co/Noticias/SiteAssets/Paginas/SIREVA-II-Colombia-2003-2012/SIREVA%20II%20Colombia%202003-2012.pdf. Accessed 8 July 2014.
6. Read RC. (2014) *Neisseria meningitidis*; clones, carriage, and disease. Clin Microbiol Infect 20:391–5. doi: 10.1111/1469-0691.12647 PMID: 24766477
7. Claus H, Maiden MC, Wilson DJ, McCarthy ND, Jolley KA, et al. (2005) Genetic analysis of meningococci carried by children and young adults. J Infect Dis 191:1263–71. PMID: 15776372
8. Taha M. (2000) Simultaneous approach for nonculture PCR-based identification and serogroup prediction of *Neisseria meningitidis*. J Clinl Microbiol 38:855–7.
9. Taha M, Fox A. (2007) Quality assessed nonculture techniques for detection and typing of meningococci. FEMS Microbiol Rev 3: 137–42.
10. Claus H, Maiden MC, Maag R, Frosch M, Vogel Ul. (2002) Many carried meningococci lack the genes required for capsule synthesis and transport. Microbiology 48:1813–9.
11. Wedege E, Hoiby EA, Rosenqvist E, Froholm O. (1990) Serotyping and subtyping of *Neisseria meningitidis* isolates by coagglutination, dot blotting and ELISA. J Med Microbiol 31:195–201. PMID: 2107317
12. Maiden MC, Bygraves JA, Feil E, Morelli G, Russell JE, Urwin M, et al. (1998) Multilocus sequence typing: a portable approach to the identification of clones within populations of pathogenic microorganisms. Proc Natl Acad Sci U S A 95:3140–5. PMID: 9501229
13. Soriano-Gabarró M, Wolter J, Hogea C, Vyse A. (2011) Carriage of *Neisseria meningitidis* in Europe: a review of studies undertaken in the region. Expert Rev Anti Infect Ther 9:761–74. doi: 10.1586/eri.11.89 PMID: 21905785
14. Trotter CL, Greenwood BM. (2007) Meningococcal carriage in the African meningitis belt. Lancet Infect Dis 7:797–803. PMID: 18045562
15. Rodriguez P, Alvarez I, Torres MT, Diaz J, Bertoglia MP, Carcamo M, et al. (2014) Meningococcal carriage prevalence in university students, 1824 years of age in Santiago, Chile. Vaccine 32:6577–80. doi: 10.1016/j.vaccine.2014.08.015

16. Espinosa de los Monteros LE, Aguilar-Ituarte F, Jiménez-Rojas LV, Kuri P, Rodríguez-Suárez RS, Gómez-Barreto D. (2009) Prevalence of *Neisseria meningitidis* carriers in children under five years of age and teenagers in certain populations of Mexico City. Salud Publica Mex 51:114–8. PMID: 19377737

17. Christensen H, May M, Bowen L, Hickman M, Trotter CL. (2010) Meningococcal carriage by age: a systematic review and meta-analysis. Lancet Infect Dis 10:853–61. doi: 10.1016/S1473-3099(10)70251-6 PMID: 21075057

18. MacLennan J, Kafatos G, Neal K, Andrews N, Cameron JC, Roberts R, et al. (2006) Social behavior and meningococcal carriage in British teenagers. Emerg Infect Dis 12:950–7. PMID: 16707051

19. Ala'aldeen DA, Oldfield NJ, Bidmos FA, Abouseada NM, Ahmed NW, Turner DP, et al. (2011) Carriage of meningococci by university students, United Kingdom. Emerg Infect Dis 17:1762–3. doi: 10.3201/eid1709.101762 PMID: 21888817

20. Bilukha OO, Rosenstein N; National Center for Infectious Diseases, Centers for Disease Control and Prevention (CDC). (2005) Prevention and control of meningococcal disease. Recommendations of the Advisory Committee on Immunization Practices (ACIP). MMWR Recomm Rep 54:1–21.

21. Centers for Disease Control and Prevention (CDC) Advisory Committee on Immunization Practices. (2007) Revised recommendations of the Advisory Committee on Immunization Practices to Vaccinate all Persons Aged 11–18 Years with Meningococcal Conjugate Vaccine. MMWR Morb Mortal Wkly Rep 56:794–5. PMID: 17694617

22. Harrison LH, Dwyer DM, Maples CT, Billmann L. (1999) Risk of meningococcal infection in college students. JAMA 281:1906–10. PMID: 10349894

23. Esposito S, Zampiero A, Terranova L, Montinaro V, Peves Rios W, Scala A, et al. (2013) Comparison of posterior pharyngeal wall and nasopharyngeal swabbing as a means of detecting the carriage of *Neisseria meningitidis* in adolescents. Eur J Clin Microbiol Infect Dis 32:1129–33. doi: 10.1007/s10096-013-1856-2 PMID: 23504236

24. Stanwell-Smith RE, Stuart JM, Hughes AO, Robinson P, Griffin MB, Cartwright K. (1994) Smoking, the environment and meningococcal disease: a case control study. Epidemiol Infect 112:315–28. PMID: 8150006

25. Tayal SC, Rashid S, Muttu KM, Hildreth AJ. (1997) Meningococcal carriage: prevalence and sex-related risk factors. J Infect 34:101–5. PMID: 9138131

26. Lourenço MC, Reis RS, Andrade AC, Tuyama M, Barroso DE. (2006) Subclinical infection of the genital tract with *Neisseria meningitidis*. Braz J Infect Dis 10:154–5. PMID: 16878269

27. Urra E, Alkorta M, Sota M, Alcalá B, Martínez I, Barrón J, et al. (2005) Orogenital transmission of *Neisseria meningitidis* serogroup C confirmed by genotyping techniques. Eur J Clin Microbiol Infect Dis 24:51–3. PMID: 15599785

28. Kristiansen PA, Diomandé F, Wei SC, Ouédraogo R, Sangaré L, Sanou I, et al. (2011) Baseline meningococcal carriage in Burkina Faso before the introduction of a meningococcal serogroup A conjugate vaccine. Clin and Vaccine Immun 18:435–43.

29. Trotter CL, Greenwood BM. (2007) Meningococcal carriage in the African meningitis belt. Lancet Infect Dis 7:797–803. PMID: 18045562

30. Leimkugel J, Hodgson A, Forgor AA, Pflüger V, Dangy JP, Smith T, et al. (2007) Clonal waves of *Neisseria* colonisation and disease in the African meningitis belt: eight- year longitudinal study in northern Ghana. PLoS Med; 4:e101. PMID: 17388665

31. Agudelo CI, Sanabria OM, Ovalle MV. (2008) Serogroup Y meningococcal disease, Colombia. Emerg Infect Dis 14:990–1. doi: 10.3201/eid1406.071357 PMID: 18507927

32. Abad R, Agudelo CI, Brandileone MC, Chanto G, Gabastou JM, Hormazabal JC, et al. (2009) Molecular characterization of invasive serogroup Y *Neisseria meningitidis* strains isolated in the Latin America region. J Infect 59:104–14. doi: 10.1016/j.jinf.2009.06.001 PMID: 19576638

33. Yazdankhah SP, Kriz P, Tzanakaki G, Kremastinou J, Kalmusova J, Musilek M, et al. (2004) Distribution of serogroups and genotypes among disease-associated and carried isolates of *Neisseria meningitidis* from the Czech Republic, Greece, and Norway. J Clin Microbiol 42:5146–53 PMID: 15528708

34. Chang Q, Tzeng YL, Stephens DS. (2012) Meningococcal disease: changes in epidemiology and prevention. Clin Epidemiol 4:237–45. doi: 10.2147/CLEP.S28410 PMID: 23071402

35. Jounio U, Saukkoriipi A, Bratcher HB, Bloigu A, Juvonen R, Silvennoinen-Kassinen S, et al. (2012) Genotypic and phenotypic characterization of carriage and invasive disease isolates of *Neisseria meningitidis* in Finland. J Clin Microbiol 50:264–73. doi: 10.1128/JCM.05385-11 PMID: 22135261

36. Climent Y, Yero D, Martinez I, Martín A, Jolley KA, Sotolongo F, et al. (2010) Clonal distribution of disease-associated and healthy carrier isolates of *Neisseria meningitidis* between 1983 and 2005 in Cuba. J Clin Microbiol 48:802–10. doi: 10.1128/JCM.01653-09 PMID: 20042619

37. Sáfadi MA, Carvalhanas TR, Paula de Lemos A, Gorla MC, Salgado M, Fukasawa LO, et al. (2014) Carriage rate and effects of vaccination after outbreaks of serogroup C meningococcal disease, Brazil, 2010. Emerg Infect Dis 20:806–11. doi: 10.3201/eid2005.130948 PMID: 24751156

38. Beddek AJ, Li MS, Kroll JS, Jordan TW, Martin DR. (2009) Evidence for capsule switching between carried and disease-causing *Neisseria meningitidis* strains. Infect Immun 77:2989–94. doi: 10.1128/IAI.00181-09 PMID: 19451248

39. von Gottberg A, du Plessis M, Cohen C, Prentice E, Schrag S, de Gouveia L, et al. (2008) Emergence of endemic serogroup W135 meningococcal disease associated with a high mortality rate in South Africa. Clin Infect Dis 46:377–86. doi: 10.1086/525260 PMID: 18181736

40. Abad R, López EL, Debbag R, Vázquez JA. (2014) Serogroup W meningococcal disease: global spread and current affect on the Southern Cone in Latin America. Epidemiol Infect. 142:2461–70. doi: 10.1017/S0950268814001149 PMID: 24831052

41. Sorhouet-Pereira C, Efron A, Gagetti P, Faccone D, Regueira M, Corso A, et al. (2013) Phenotypic and genotypic characteristics of *Neisseria meningitidis* disease-causing strains in Argentina, 2010. PLoS One; 8(3):e58065. doi: 10.1371/journal.pone.0058065 PMID: 23483970

42. Araya P, Fernández J, Del Canto F, Seoane M, Ibarz-Pavón AB, Barra G, et al. (2015) Neisseria meningitidis ST-11 Clonal Complex, Chile 2012. Emerg Infect Dis. 21:339–41. doi: 10.3201/eid2102.140746 PMID: 25625322
